# Graphene-based tunable hyperbolic microcavity

**DOI:** 10.1038/s41598-020-80022-9

**Published:** 2021-01-08

**Authors:** Michał Dudek, Rafał Kowerdziej, Alessandro Pianelli, Janusz Parka

**Affiliations:** Institute of Applied Physics, Military University of Technology, 2 Kaliskiego St., 00-908 Warsaw, Poland

**Keywords:** Optics and photonics, Graphene, Nanoscale materials, Composites

## Abstract

Graphene-based hyperbolic metamaterials provide a unique scaffold for designing nanophotonic devices with active functionalities. In this work, we have theoretically demonstrated that the characteristics of a polarization-dependent tunable hyperbolic microcavity in the mid-infrared frequencies could be realized by modulating the thickness of the dielectric layers, and thus breaking periodicity in a graphene-based hyperbolic metamaterial stack. Transmission of the tunable microcavity shows a Fabry–Perot resonant mode with a Q-factor > 20, and a sixfold local enhancement of electric field intensity. It was found that by varying the gating voltage of graphene from 2 to 8 V, the device could be self-regulated with respect to both the intensity (up to 30%) and spectrum (up to 2.1 µm). In addition, the switching of the device was considered over a wide range of incident angles for both the transverse electric and transverse magnetic modes. Finally, numerical analysis indicated that a topological transition between elliptic and type II hyperbolic dispersion could be actively switched. The proposed scheme represents a remarkably versatile platform for the mid-infrared wave manipulation and may find applications in many multi-functional architectures, including ultra-sensitive filters, low-threshold lasers, and photonic chips.

## Introduction

The introduction of artificially engineered composite materials that function as electromagnetic metamaterials has allowed for a novel light-matter interaction at subwavelength scales^1,2^. Metamaterials can be realized via different architectures having been designed and manipulated to realize exotic effects arising from their structure sequence and configuration. Recently, the attention in metamaterial research has primarily been directed towards tunable and reconfigurable structures that can be used for an active wavefront shaping. This can serve as an effective tool for regulation and switching of electromagnetic waves spreading in multifunctional structured devices^3–10^. Active metamaterial functionalities can be obtained using liquid crystals^11–14^, superconductors^15,16^, nonlinear media^17,18^, and carrier injection schemes^19,20^. Nonetheless, despite the numerous available methods, studies on active graphene-based metamaterials are of a particular importance^21,22^. This is because their properties can be reversibly switched by changing the chemical potential of graphene sheets via electric biasing or optical pumping. Among the different classes of metamaterials, graphene-based hyperbolic metamaterials (GHMMs) have gained considerable attention because of their unique hyperbolic isofrequency, i.e., their permittivity and permeability tensor elements (along the principal axes) are of opposite signs, resulting in a strong anisotropy, support for propagating high *k*-modes, and an enhanced photonic density of states^23–32^. Graphene, as a completely two-dimensional (2D) material that only conducts in the plane, shows an anisotropicity that is appropriate for hyperbolic metamaterials (HMMs). Considering an extraordinary wave (TM polarized) in a uniaxial medium, the isofrequency relation can be stated as $\frac{{k_{x}^{2} + k_{y}^{2} }}{{\varepsilon_{ \bot } }} + \frac{{k_{z}^{2} }}{{\varepsilon_{||} }} = \left( {\frac{\omega }{c}} \right)^{2}$, where the wave vector of a propagating wave is given by $\vec{k} = \left[ {k_{x} ,k_{y} , k_{z} } \right]$, *ω* is the wave frequency, and *c* is the speed of light^2,31^. Hence, such a medium has a dielectric response defined by a tensor ${\vec{\upvarepsilon }} = \left[ {\upvarepsilon _{x} ,\upvarepsilon _{y} ,\upvarepsilon _{z} } \right]$, where the in-plane isotropic components are *ε*_*x*_ = *ε*_*y*_ = *ε*_∥_ and the out-of-plane component is *ε*_*z*_ = *ε*_⊥_. The spherical isofrequency surface of the vacuum distorts to an ellipsoid for the anisotropic case. However, when extreme anisotropy is present, such that *ε*_∥_ ⋅ *ε*_⊥_ < 0, the isofrequency surface opens into an open hyperboloid^2^. Thereby, the same material may affect the incident radiation as a metal or as a dielectric depending on the orientation of the wavevector $\vec{k}$. This differs from the behavior of most standard materials, where the isofrequency surface forms an ellipsoid or sphere. The HMMs were initially designed to overcome the diffraction limit of optical imaging. Shortly after, they were observed to exhibit a number of unusual phenomena including Purcell factor enhancement^33^, intensified spontaneous emission^34^, negative refraction^35^, strengthened superconductivity^36^, and increased reflection^23^. Importantly, HMMs are most commonly created with two-structure classes such as metal-dielectric multilayers^23–29^ and metallic nanorod arrays^37^. In the case of GHMMs, graphene provides conducting electrons making the extreme anisotropicity achievable. As the thinnest material available, graphene also functions as a perfect building slab for multilayer systems, as it enables the minimum possible period and, consequently, the highest possible cutoff for the high k-modes; this has been limited in semiconductor and metal-based HMMs by the high thickness of those materials^30^. The unusual effects mentioned above have increased a significant amount of theoretical studies focused on the utilization of graphene as an active constituent of HMMs^28,29^. Nevertheless, the reported properties relate mainly to the potential of the active switching hyperbolic properties of graphene/dielectric planar heterostructures. Additionally, studies investigating the use of GHMMs as effective photonic devices remain insufficient; therefore, a further progress is necessary.

In this work, we report a polarization-dependent tunable microcavity based on a GHMM operating in the mid-infrared (mid-IR) frequencies. The operating principle of a tunable device was based on the thickness modulation of the dielectric layers in a GHMM stack. Thereby, breaking the periodicity in a GHMM is manifested in cavity resonant modes. We have modeled a stack consisting of 20-unit cells in which gaps between the graphene sheets are supposed to be filled with a dielectric of varying thickness described by a triangle wave function. It was shown that a subwavelength confinement attributed to volume plasmon polaritons can be optimized and efficiently controlled by acting both on the base dielectric thickness that we modulate and on the gate voltage of graphene. Our calculations proved that the obtained microcavity can be reversibly tuned with respect to both intensity (up to 30%) and spectrum (up to 2.1 µm) by varying the voltage-dependent sheet conductivity of graphene from 2 to 8 V; thus, adjusting the Fermi level via the chemical potential allowing for an ultrafast electrical modulation. Additionally, switching of the device was tested over a wide range of incident angles for both transverse electric and transverse magnetic (TE/TM) modes. Finally, determination of both the in-plane isotropic components *ε*_*x*_ = *ε*_*y*_ = *ε*_∥_ and the out-of-plane component *ε*_*z*_ = *ε*_⊥_ allowed for determination of the topological transition between elliptic and type II hyperbolic dispersion.

## Numerical model

**Figure 1:**
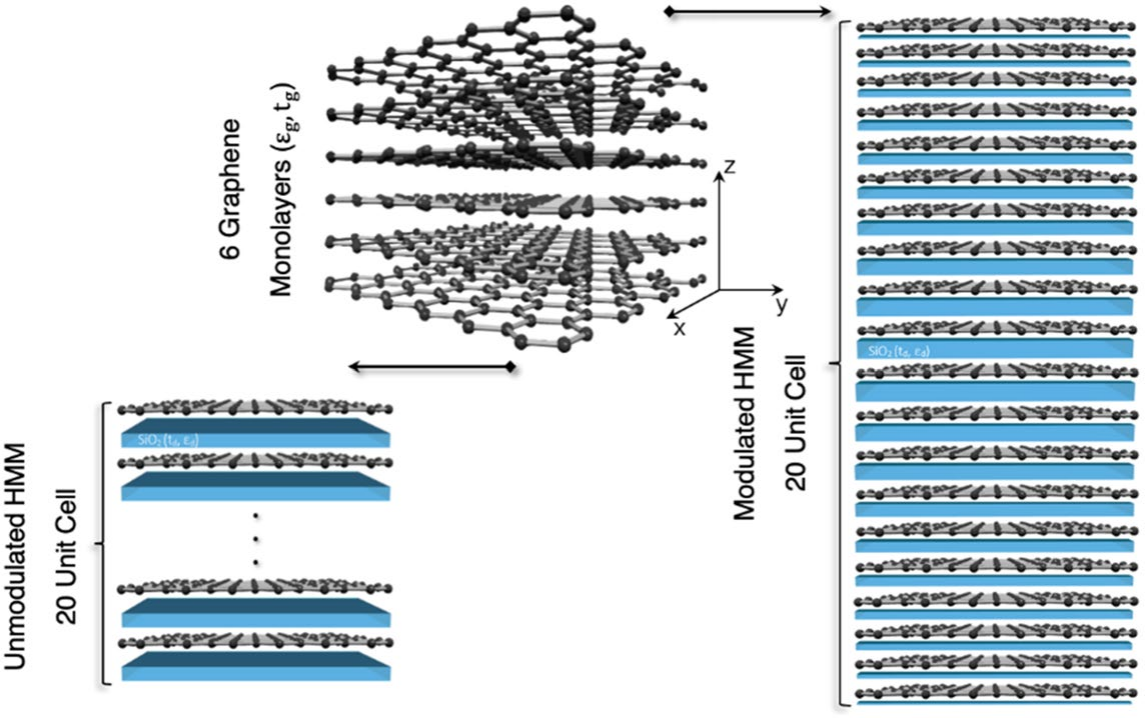
3D scheme for both the unmodulated and modulated composite HMMs.

Full-wave numerical simulations of the tunable graphene-based microresonator were performed using Lumerical FDTD Solutions software which employs an algorithm based on the conformal finite-difference time-domain method. The modeled HMM geometry shown in Fig. 1 is built of $N$ = 20 unit cells that are based on alternating subwavelength layers of graphene and silica, which are characterized by thicknesses $t_{g}$, $t_{d}$ and permittivities $\varepsilon_{g}$_,_ $\varepsilon_{d}$, respectively. Recently, our group demonstrated that with an increasing number of graphene monolayers in the HMM unit cell, the edge filter characteristics became more efficient^23^. Based on our recent studies, here, we set this value to a constant $N_{g} =$ 6 as the optimal value for the mid-IR^23^, and the thickness of the graphene monolayer was set to $t_{g}$ = 0.35 nm.

The effective medium permittivities for an anisotropic multilayer composite were computed using the effective medium theory (EMT). The method follows a generalized Maxwell–Garnett approach to obtain analytical expressions for the effective permittivity in parallel (||) and perpendicular (⊥) directions defined below for the multilayer metamaterial^31^:

1 $$ \varepsilon_{\parallel } = \frac{{t_{g} \varepsilon_{g} + t_{d} \varepsilon_{d} }}{{t_{g} + t_{d} }}, $$

2 $$ \varepsilon_{ \bot } = \frac{{\varepsilon_{g} \varepsilon_{d} \left( {t_{g} + t_{d} } \right)}}{{t_{g} \varepsilon_{d} + t_{d} \varepsilon_{g} }}. $$

If we assume that the electronic band structure of a graphene sheet cannot be affected by the neighboring graphene sheets, the effective permittivity of graphene $\varepsilon_{g}$ can be written based on the formula given by Falkovsky and Varlamov^38^:

3 $$ \varepsilon_{g} = 1 + i\frac{{\sigma \left( {\omega ,\Gamma ,\mu_{c} ,T} \right)}}{{\omega \varepsilon_{0} t_{g} }}, $$

where $\varepsilon_{0}$ is the permittivity of the vacuum and $\sigma$ is the conductivity of a graphene monolayer. The graphene surface conductivity can be computed by adopting the Kubo formula^39,40^. Without taking into account the external magnetic field, the isotropic surface conductivity $\sigma$ of graphene can be expressed as the sum of the intra-band $\sigma$_intra_ and the inter-band term $\sigma$_inter_:

4 $$ \sigma_{intra} \left( {\omega ,\Gamma ,\mu_{c} ,T} \right) = \frac{{ - ie^{2} }}{{\pi \hbar^{2} \left( {\omega + i2\Gamma } \right)}}\mathop \smallint \limits_{0}^{\infty } \xi \left( {\frac{{\partial f_{d} \left( \xi \right)}}{\partial \xi } - \frac{{\partial f_{d} \left( { - \xi } \right)}}{\partial \xi }} \right)d\xi , $$

5 $$ \sigma_{inter} \left( {\omega ,\Gamma ,\mu_{c} ,T} \right) = \frac{{ie^{2} \left( {\omega + i2\Gamma } \right)}}{{\pi \hbar^{2} }}\mathop \smallint \limits_{0}^{\infty } \frac{{f_{d} \left( { - \xi } \right) - f_{d} \left( \xi \right)}}{{\left( {\omega + i2\Gamma } \right)^{2} - 4\left( {\xi /\hbar } \right)^{2} }}d\xi , $$

where $\omega$ is the angular frequency of the incident electromagnetic wave, $\Gamma$ is the scattering rate that is set as 0.1 meV, $\mu_{c}$ is the chemical potential, $T$ is the temperature, $e$ is the electron charge, $\hbar$ is the reduced Plank constant, $k_{B}$ is the Boltzmann constant, and $f_{d} \left( \xi \right)$ is the Fermi–Dirac function: $f_{d} \left( \xi \right) = \left[ {exp\left( {\xi - \mu_{c} /k_{B} T} \right) + 1} \right]^{ - 1}$ which gives the feasibility that a given available electron energy state will be engaged at a given temperature^1,5^. The chemical potential ($\mu_{c}$) can be determined from the following equation^22^:

6 $$ \left| {\mu_{c} } \right| = \hbar v_{F} \sqrt {\pi \left| {a_{0} \left( {V_{g} - V_{D} } \right)} \right|} , $$

where $v_{F}$ is the Fermi velocity in graphene (~ 10^6^ m/s), $a_{0}$ = 9∙10^16^ m^−2^ V^−1^ is an empirical constant, and $V_{D}$ is the offset bias voltage, which in our model was assumed to be 0 V, and can be electrically controlled by an applied gate voltage $V_{g}$^21^. In the sub-wavelength limit, the graphene-dielectric layered architecture can be examined as a homogeneous effective medium with the anisotropic permittivity tensor ${\vec{\upvarepsilon }} = \left[ {\upvarepsilon _{x} ,\upvarepsilon _{y} ,\upvarepsilon _{z} } \right]$. Considering the Cartesian coordinate system depicted in Fig. 1, the following expressions occur:$\varepsilon_{x} = \varepsilon_{y} = f_{g} \varepsilon_{g} + f_{d} \varepsilon_{d}$, $\varepsilon_{z} = \left( {\frac{{f_{g} }}{{\varepsilon_{g} }} + \frac{{f_{d} }}{{\varepsilon_{d} }}} \right)^{ - 1} ,$ where $f_{g} = \frac{{t_{g} }}{t}$ and $f_{d} = \frac{{t_{d} }}{t}$ are the filling ratios of the graphene sheet and dielectric, respectively. Importantly, when $t_{g} \ll t_{d}$, the following relationship exists: $\varepsilon_{x} = \varepsilon_{y} \approx \varepsilon_{d} + i\frac{{\sigma \left( {\omega ,\Gamma ,\mu_{c} ,T} \right)}}{{\omega \varepsilon_{0} t_{g} }}$. This reflects the averaging of the effective displacement current (including both the displacement current in the silica and conduction current in the monolayer graphene) over the related electric field in a unit cell of GHMM^32^.

In the proposed model, the dielectric permittivity was set to a constant $\varepsilon_{d}$ = 2.1025, based on the SiO_2_ averaged material data. To design the GHMM-based microcavity, the thickness of the dielectric layers in the HMM stack was modulated. The modulation was based on a half period triangle wave function, and the base dielectric thickness in the first unit cell was set as $t_{d} \left( 1 \right) = t_{0}$. Therefore, the modulation of the dielectric thickness assumes the form:

7 $$ t_{d} \left( n \right) = t_{0} \left[ {1 + c_{{\Delta }} \left( n \right)} \right], $$

where $n = \left( {1 \ldots N} \right)$ is the number of consecutive unit cell, $N$ is the number of all unit cells, and the triangle wave function coefficients $c_{{\Delta }}$ were additionally rounded down to two decimal places, and may be expressed as:

8 $$ c_{{\Delta }} \left( n \right) = \frac{2}{\pi }\sin^{ - 1} \left[ {\sin \left( {\pi \frac{n - 1}{{N - 1}}} \right)} \right]. $$

It should be noted that the dielectric thickness increased until reaching a maximum value in the middle of the stack and, then, it decreased to reach the initial value again in the last unit cell.

In our analysis we used periodic boundary conditions (PBC) for normal incidence, and broadband fixed angle source technique (BFAST) boundary conditions for obtaining the angular characteristics. In addition, to ensure both stability of the algorithm and high accuracy of calculations, we used uniform spatial grid with 100 nm step and additional mesh override regions around graphene layers with 1 nm step and 10 nm buffer; the auto shutoff threshold was set as 10^−7^. The described methodology allowed us to adapt the GHMM to design a tunable hyperbolic microcavity in the mid-IR frequencies.

## Results and discussion

**Figure 2:**
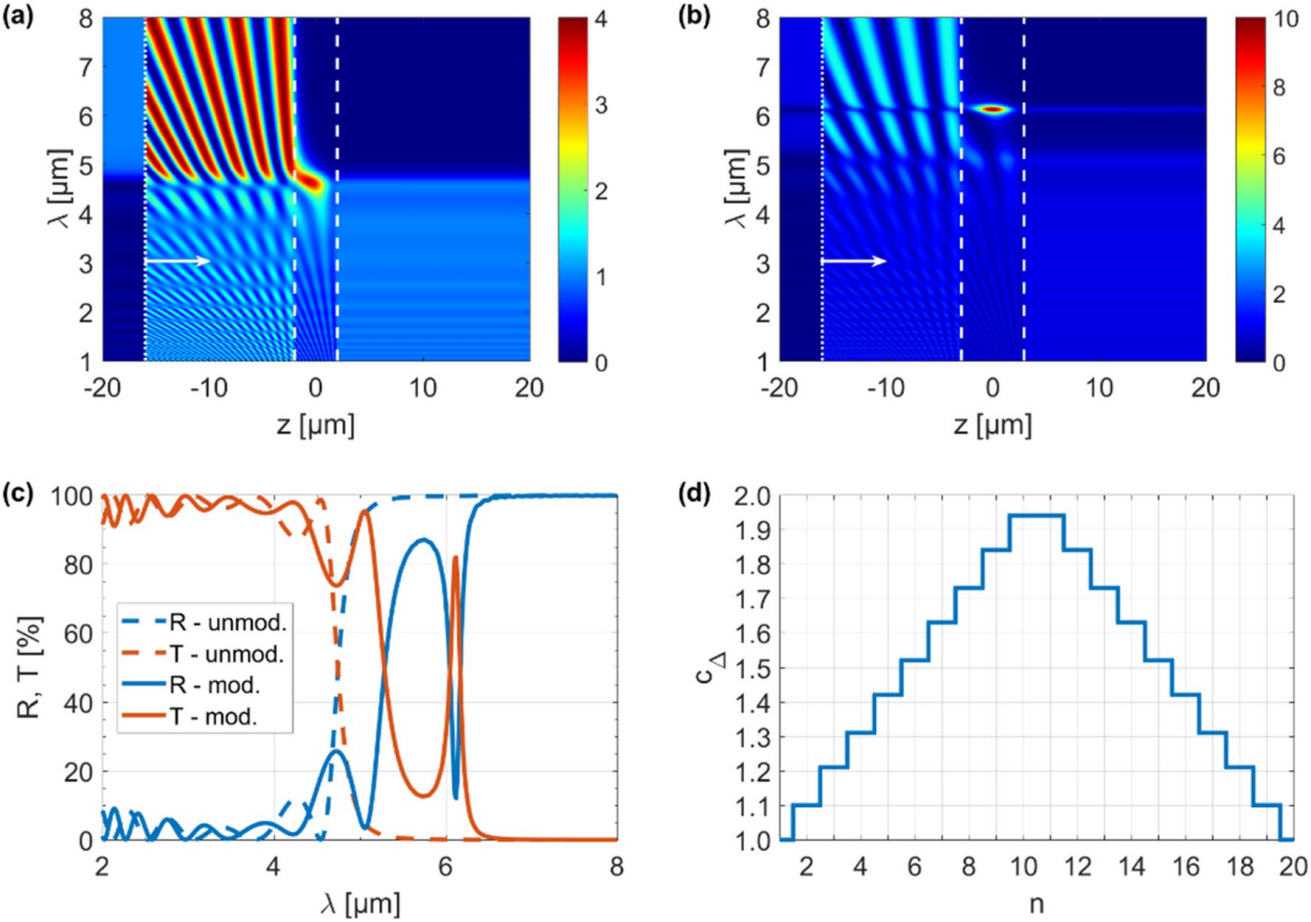
Electric field intensity for (**a**) unmodulated and (**b**) modulated structure, (**c**) their reflection and transmission comparison, and (**d**) triangle wave function coefficients used for the dielectric thickness modulation.

In order to demonstrate that modulation of the dielectric thickness in the GHMM stack results in a microcavity, we designed two stacks, i.e., a stack with an unmodulated (constant) dielectric thickness and a modulated one. Then, the spatial distribution of their electric field intensities was studied, as shown in Fig. 2a,b. The intensity maps consist of four main areas and should be interpreted as follows: starting from left side, from − 20 to − 16 μm, is the reflection area, where only the reflected field is visible. Then, from − 16 μm (source position) to the left border of stack is an interference area, where part of the field reflected from the structure interferes with the source field. Next, and most important, is the area inside the stack, whose boundaries are denoted with white dashed lines. The last section is the transmission area, where only the transmitted field is visible. It should be noted that in the case of an unmodulated structure, the dielectric thickness was constant throughout the entire stack volume and equaled $t_{d}$ = $t_{0}$ = 200 nm. In our study, the excitation was in the form of a plane wave, and its source, denoted by the white dotted line, was located at 16 μm to the left from the center of the stack, which means that the wave propagates along the *z* direction, as depicted by white arrows. In Fig. 2a, for the case of a stack with an unmodulated dielectric thickness, we could observe that the electric field intensity was low for wavelengths from 2 to ~ 4.7 μm, followed by a rapid increase, which was observed up to 8 μm. Hence, for a wavelength of 4.7 μm there is a transition from high transmission to high reflectance (see Fig. 2c); thus, the device acts as an edge filter. Furthermore, the transition from high transmission to high reflectance coincides with a change in the isofrequency dispersion regime from elliptic to type II hyperbolic which was previously reported by our group^23^. In addition, the wavelength at which this transition occurs can be self-regulated via a voltage-controlled chemical potential. Then, using the procedure described above, we modulated the dielectric thickness in the HMM stack with a triangle wave function for $t_{0}$ = 200 nm and again analyzed the intensity of the electric field, as shown in Fig. 2b. For the HMM with a modulated dielectric thickness we observed a previously unreported effect. Interestingly, at a wavelength of ~ 6.1 μm, an additional resonance peak appeared and the transmission was significantly amplified (see Fig. 2c), which means that for this wavelength the modulated stack acted as a microresonator. The enhancement coefficient for the unmodulated structure (Fig. 2a) was ~ 3.8, while it was ~ 10.6 for the modulated one (Fig. 2b). Importantly, the resonance depth can be effectively tuned via electrical excitation, which allows for control of the chemical potential of graphene. Thus, the designed architecture allowed for multifunctional applications and added an additional level of functionality. It is worth emphasizing that these results were achieved for constant bias voltage values ($V_{g}$ = 5 V for $\mu_{c}$ = 0.8 eV) and number of unit cells ($N$ = 20). The effective thicknesses $d$ for the unmodulated and modulated stacks were 4.042 μm and 5.922 μm, respectively. In our approach the modulation of the dielectric thickness was realized based on the triangle wave function described by Eq. (8) and illustrated in Fig. 2d.

An extensive explanation of the light propagation inside anisotropic multilayer composites has been given by Meier^41^ and by Avrutsky et al.^42^. Graphene-based hyperbolic microcavity supports plasmonic modes which are commonly denominated gap plasmon polaritons (GPPs) and are strongly confined inside the stack^43^. Noteworthy is the fact that when the number of adjacent nanocavities (intended as the number of dielectric slabs loaded between two graphene sheets) increases, a collective repulsion of these modal indices appears. This interplay between these cavity plasmon modes stimulates their hybridization^44^, thus giving rise to extremely confined volume modes called volume plasmon polaritons (VPPs)^45^. Indeed, when the parameters of a subwavelength graphene-based multilayer composite are modulated (or superstructured) on a larger, wavelength scale, the propagation of VPPs in the resulting multiscale hyperbolic metamaterials is accompanied by photonic-band-gap phenomena. In our case, modulation of the dielectric thickness with a triangle wave function enabled us to achieve a great degree of control over such plasmons in the entire volume of the stack. It should be noted that when the stack geometry is periodic (i.e., unmodulated), stop bands due to Bragg reflection form within the volume a plasmonic band^23,45–47^. Contrarily, when cavity layers are sandwiched in an otherwise periodic stack, resonance peak of the Fabry–Perot (FP) nature arises within the stop band. Specifically, mid-IR waves reflecting from a partially reflecting graphene separated by a distance described by the triangle wave function can interfere constructively and form a resonating FP mode. At this point, it is worth noting that instead of mirrors (used in most classic resonance cavities) as a separate section, interface of dielectric/magnetic materials can act as a reflecting surface depending on the strength of dielectric permittivity (ε_r_) and magnetic permeability (μ_r_), and it can support FP modes in varieties of dielectric resonators^48^. A rectangular bar of silicon nanowire and dielectric nano ribbons are selected examples for supporting this statement^49,50^.

**Figure 3:**
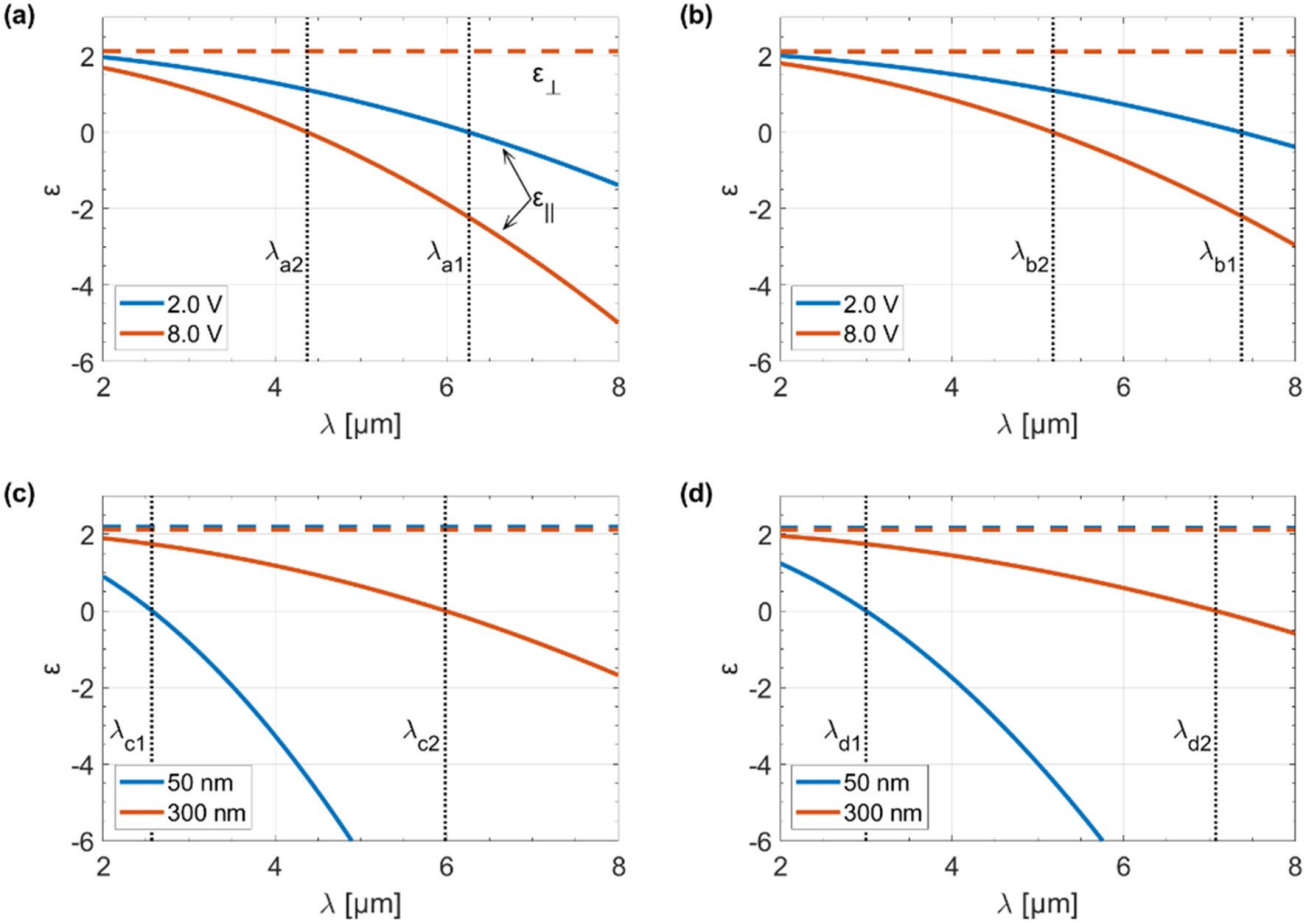
Real part of the permittivity tensor components as a function of a wavelength for different gate voltage ($V_{g}$) for (**a**) unmodulated and (**b**) modulated structure and for different base dielectric thickness ($t_{0}$) for (**c**) unmodulated and (**d**) modulated structure. Parallel permittivity components are denoted with solid lines while perpendicular components are denoted with dashed lines.

In order to determine the maximum tunability of both unmodulated and modulated GHMM, based on the method described above, we determined real part of the permittivity tensor components as a function of a wavelength for two extreme voltages ($V_{g}$ = 2 V and $V_{g}$ = 8 V) and for the minimum (50 nm) and maximum (300 nm) base dielectric thickness $(t_{0} )$, as shown in Fig. 3. Besides, taking into account the resonance frequencies (i.e., wavelengths for which individual components of the effective diagonal tensor are equal to zero), we found the transition wavelengths from elliptic ($\varepsilon_{\parallel } > 0$ and $\varepsilon_{ \bot } > 0$) to type II hyperbolic dispersion ($\varepsilon_{\parallel } < 0$ and $\varepsilon_{ \bot } > 0$), as illustrated in Table 1. The considered structure manifests such behavior due to the careful choice of its fundamental building blocks. In Fig. 3a,b, when the gate voltage varies between V_g_ = 2 V and V_g_ = 8 V, the transition from elliptic to type II hyperbolic dispersion can be tuned effectively up to ~ 1.9 μm for unmodulated and up to ~ 2.2 μm for modulated GHMM. Furthermore, by modifying the base dielectric thickness $(t_{0} )$ from 50 to 300 nm we are able to tune the transition from elliptic to type II hyperbolic dispersion up to ~ 3.4 μm for unmodulated and up to ~ 4.1 μm for modulated structure, as illustrated in Fig. 3c,d. It is worth emphasizing that for each case under consideration, the perpendicular permittivity tensor component (*ε*_⊥_) is constant while only the parallel permittivity component (*ε*_∥_) changes with wavelength. Interestingly, the change in the gate voltage of graphene does not affect the perpendicular permittivity of the HMM and, therefore, the dashed lines in Fig. 3a,b coincide. This behavior is consistent with the properties of graphene as a 2D material. Besides, it is worth noting that the resonance frequencies depend on the Fermi velocity, i.e. the lower the Fermi velocity, the higher the resonant frequency. Importantly, the desired resonant frequency can be obtained by adjusting the voltage.

**Table 1:** Transition wavelengths from elliptic to type II hyperbolic dispersion based on plots shown in Fig. 3.

Gate voltage ($V_{g}$) (V)	Base dielectric thickness ($t_{0}$) (nm)	Transition wavelength	
		Unmodulated structure	Modulated structure
2.0	200	6.260 μm (λ_a1_)	7.376 μm (λ_b1_)
8.0	200	4.376 μm (λ_a2_)	5.180 μm (λ_b2_)
5.0	50	2.564 μm (λ_c1_)	2.996 μm (λ_d1_)
5.0	300	5.984 μm (λ_c2_)	7.076 μm (λ_d2_)

**Figure 4:**
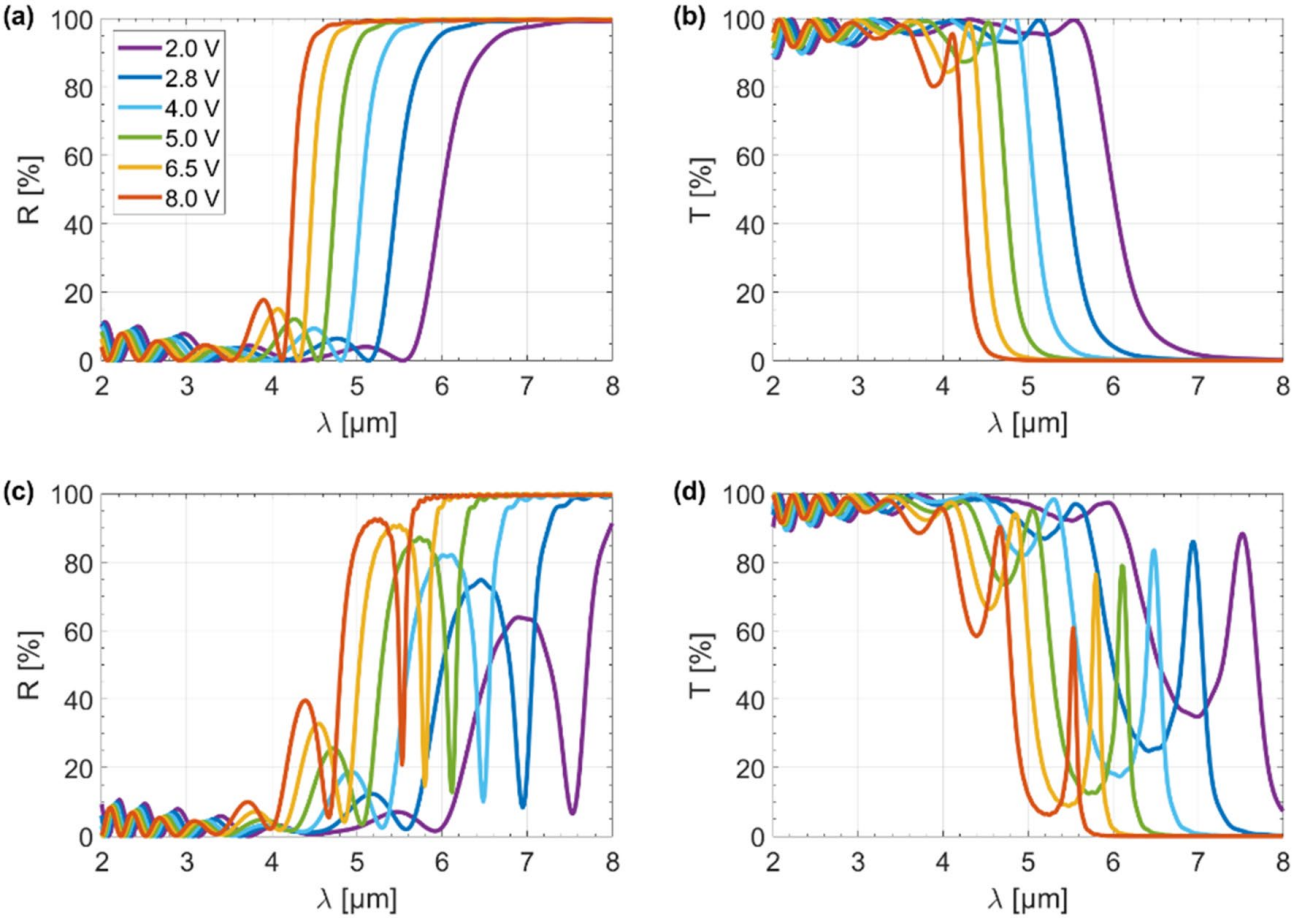
Reflection (**a**) and transmission (**b**) spectra for the unmodulated structure, and reflection (**c**) and transmission (**d**) spectra for the modulated structure as a function of gate voltage ($V_{g}$).

To fully explain the switching mechanism of the hyperbolic microcavity, we examined the effect of an external electrical excitation on the tunability of both the transmission and reflection as a function of wavelength, as shown in Fig. 4a–d. Calculations were made for a stack with a constant dielectric thickness ($t_{d}$ = $t_{0}$ = 200 nm) and a stack where the dielectric thickness distribution in individual layers is described by the triangle wave function ($t_{0}$ = 200 nm) shown in Fig. 2d. For the unmodulated structure, it can be seen that the reflectance increased rapidly and was accompanied by a sharp decrease in transmittance (Fig. 4a,b). Therefore, the unmodulated HMM works like an efficient edge filter^23^. Additionally, as the gate voltage gradually increased from $V_{g}$ = 2 to 8 V, the reflectance blue-shifted by up to 2.1 µm. It should be noted that this process was totally reversible, i.e., when the gate voltage decreases from $V_{g}$ = 8 to 2 V, the reflectance red-shifted by up to 2.1 µm. From our previous results, we can conclude that the transition from high transmission to high reflectance coincides with a change of the isofrequency dispersion regime from elliptic to type II hyperbolic^23^. Hence, the type II hyperbolic dispersion can be self-regulated by up to 2.1 µm by varying the applied voltage, and thus the chemical potential of graphene can be adjusted. The main difference between the results for stacks with unmodulated and modulated dielectric thicknesses was the presence of a narrowband Fabry–Perot resonance peak which was manifested as a sharp decrease in the reflection and a simultaneous sharp increase in the transmission, as evident from Fig. 4c,d. Importantly, the peak can be blue/red-shifted by tuning the gating voltage of the graphene sheets between $V_{g}$ = 2 to 8 V with respect to both the intensity (up to 30%) and spectrum (up to 2.1 µm), as illustrated in Fig. 4c,d. Interestingly, the greater the external electrical stimulus, the greater the Fabry–Perot resonance depth. This is because higher voltage results in a more efficient generation of VPPs, the importance of which we have already mentioned. It should be noted that a change in voltage from $V_{g}$ = 2 to 8 V in our study corresponds to a change in the chemical potential of graphene from $\mu_{c}$ = 0.5 to 1.0 eV.

**Figure 5:**
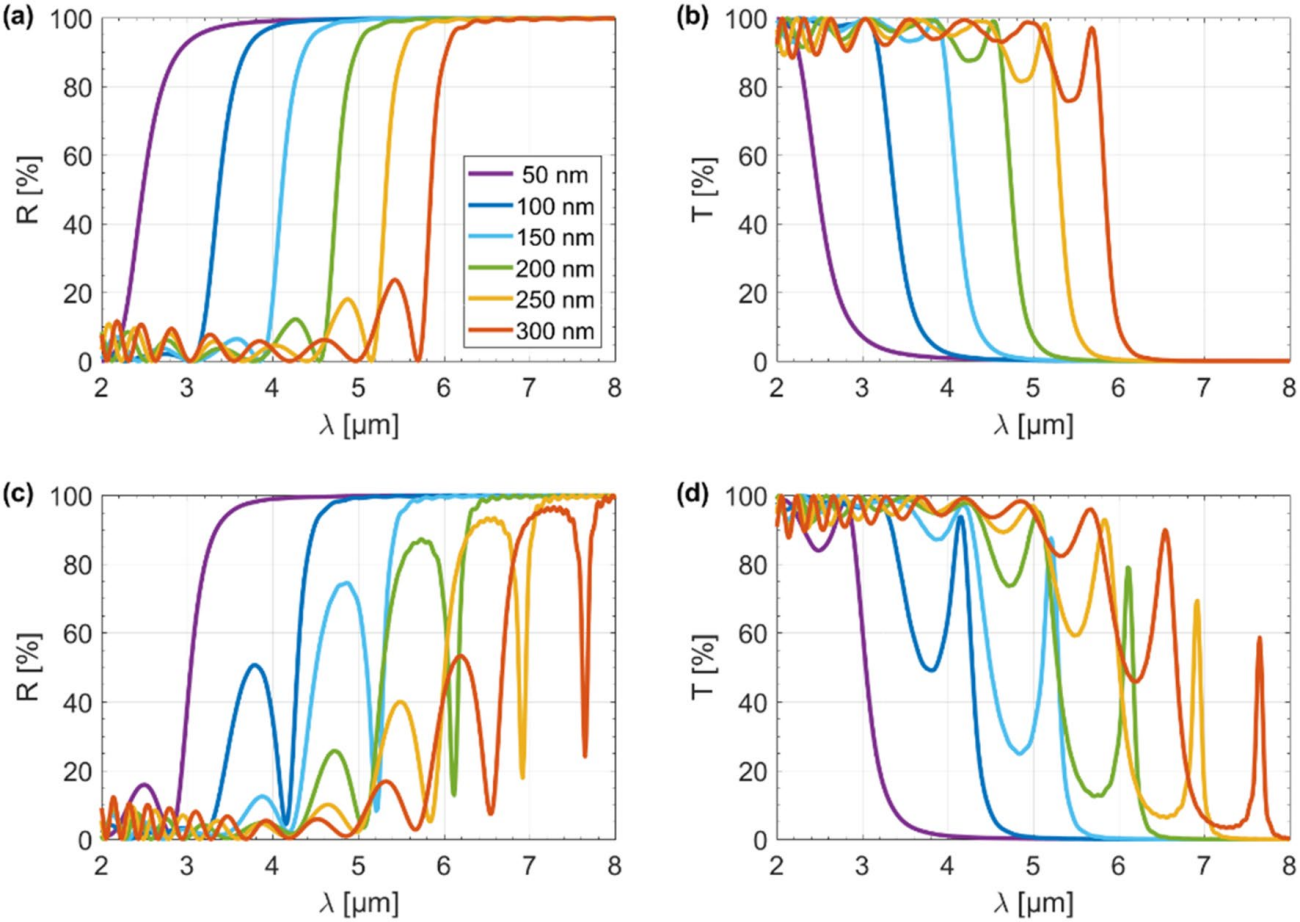
Reflection (**a**) and transmission (**b**) spectra for the unmodulated structure and reflection (**c**) and transmission (**d**) spectra for the modulated structure as a function of the base dielectric thickness ($t_{0}$).

To determine the optimal architecture for the hyperbolic microcavity, we also investigated the effect of the base dielectric thickness on the transmission/reflection spectra for both the unmodulated and modulated structures, as shown in Fig. 5a–d. Importantly, these results were obtained for a constant gate voltage value ($V_{g}$ = 5 V for $\mu_{c}$ = 0.8 eV). For a stack built on the basis of a constant dielectric thickness, by increasing thickness from 50 nm up to 300 nm, we were able to shift the transition between the high reflection and high transmission states by up to 3.4 µm, as confirmed by Fig. 5a,b. Thus, the elliptic/hyperbolic type II topological transition could also be tuned by 3.4 µm. However, the stack structure with the modulated dielectric thickness behaved in a completely different manner, as evident from Fig. 5c,d. In this case, as the thickness of the dielectric (which was modulated adopting triangle wave function) increased, we observe a redshift of the resonance peak. In addition, as the base thickness of the dielectric increases from 50 to 300 nm, the intensity of the resonance also increased. At the same time, the transmission of the device started to decrease because of increasing reflection and absorption. Thus, a base dielectric thickness of $t_{0}$ = 200 nm, which still had a transmission of ~ 80%, was chosen to optimize the performance of the tunable microcavity in mid-IR.

**Figure 6:**
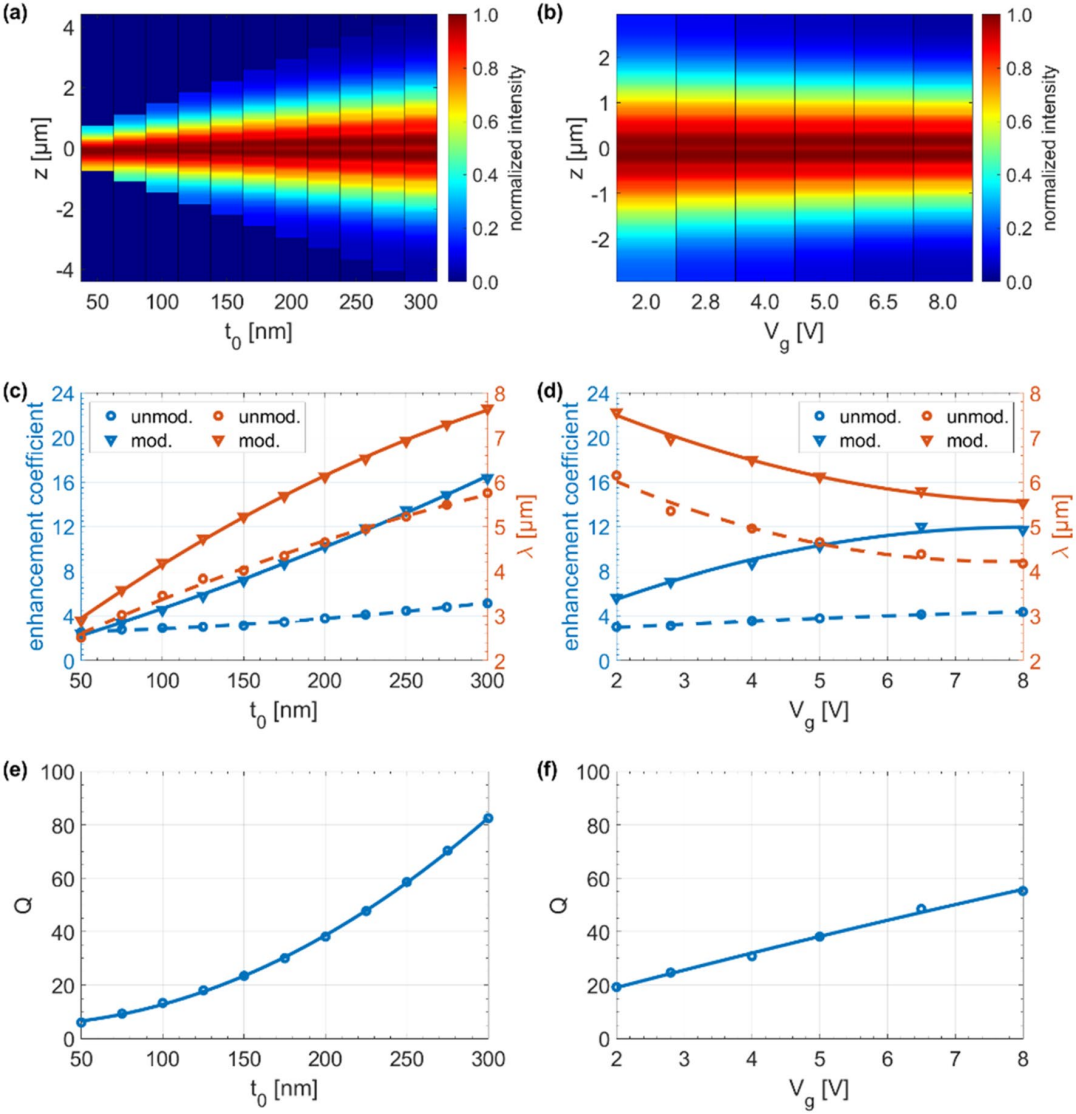
Metadevice performance; normalized electric field intensity cross-sections at resonance wavelengths as a function of (**a**) base dielectric thickness and (**b**) applied gate voltage; resonance spectral position and resonance enhancement coefficient as a function of (**c**) base dielectric thickness and (**d**) applied gate voltage; hyperbolic microcavity Q-factor as a function of (**e**) base dielectric thickness and (**f**) applied gate voltage. All curves are quadratic polynomial fits to the data.

The above-mentioned properties of the hyperbolic microcavity, i.e., enhancement of the electric field intensity, shift of the FP resonance spectral position, and microcavity Q-factor can be expressed as a function of both, the base dielectric thickness $$(t_{0} )$$ that we modulate, and the applied gate voltage (V_g_), as depicted in Fig. 6a–f. In Fig. 6a,b, we can notice that the closer to the center of the stack, the intensity of the electric field increases which is consistent with the principle of operation of a hyperbolic microcavity described above, i.e., interaction between cavity plasmon modes is the strongest in the middle of the stack, where the dielectric thickness is the largest. Next, we found that with an increase in the base thickness of the dielectric from $$t_{0}$$ = 50 to 300 nm, there was a more than six-fold increase in the enhancement coefficient for the structure with modulation of the dielectric thickness, compared with the unmodulated one that had only a two-fold increase, as evident from Fig. 6c. Furthermore, in case of the unmodulated stack, by varying the dielectric thickness between $$t_{d} $$ = 50 and 300 nm; a blue/red-shift of the transition from a high transmission to high reflection up to 2.8 µm was observed; this corresponds with a shift of the topological transition from elliptic to a type II hyperbolic dispersion also by 2.8 µm. Importantly, tunability of a FP resonance peak was also considered. In this case, the largest shift of 3.8 µm occurred for a change of the base dielectric thickness from $$t_{0}$$ = 50 to 300 nm. Moreover, the increase of the gate voltage from $$V_{g}$$ = 2 to 8 V resulted in more than a two-fold increase in the enhancement coefficient for modulated GHMM compared to only almost one and a half-fold increase for unmodulated GHMM, as shown in Fig. 6d. In order to fully characterize the hyperbolic microcavity, the quality factor (Q-factor) of the FP resonances was calculated in terms of both the change in base dielectric thickness and the applied voltage, as illustrated in Fig. 6e,f. Our results show that Q-factor is proportional to both the increase in the base dielectric thickness and the applied electrical excitation. It should be noted, that for practical applications critical is the implementation of a cavity with both high Q-factor and small modal volume (Vol). The ratio Q/Vol regulates the strength of the multiple cavity interactions, and an ultra-small cavity enables a large-scale integration and a single-mode operation for a broad range of wavelengths^51,52^. Therefore, the presented concept based on GHMMs seems to be particularly promising. Additionally, unlike classic resonant microcavities, the Q-factor increases with increasing electrical stimuli, and thus the efficiency of the device also increases. It is crucial in state-of-the-art technologies, as it gives the possibility of increasing the Q-factor and tunability at the same time.

**Figure 7:**
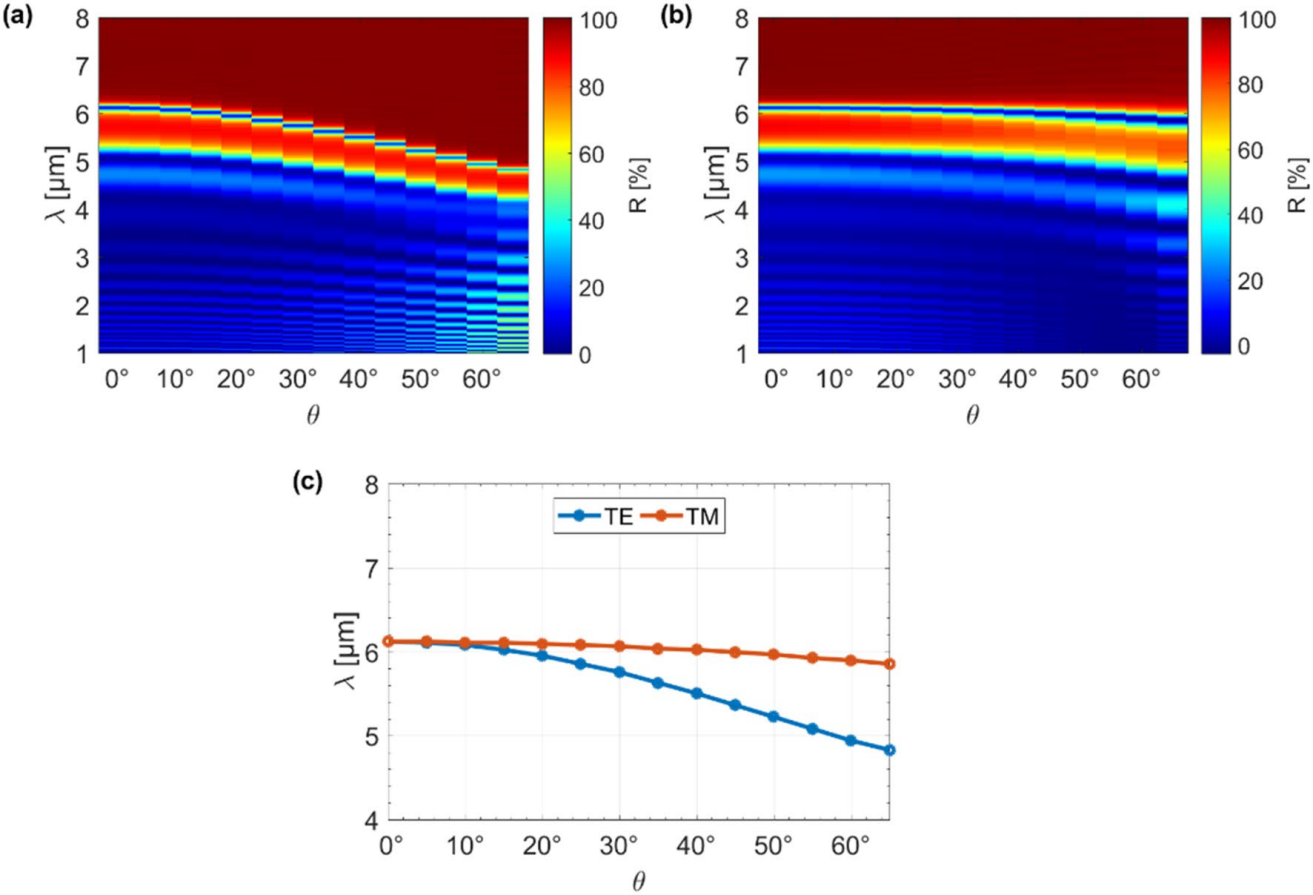
Angular reflection spectra for (**a**) TE and (**b**) TM polarized waves, together with the (**c**) reflection dip spectral position for different incidence angles.

In the last step, we studied the angular reflection characteristics, as well as the spectral position of the dip (corresponding to the FP peak in transmission) for both the TE and TM modes for the hyperbolic microcavity, as shown in Fig. 7a–c. It should be noted that the value of the chemical potential was constant at $V_{g}$ = 5 V, and the base thicknesses of the unmodulated and modulated structures were also constant and set to $t_{d} = t_{0}$ = 200 nm. It can be seen that the reflectance (and, also corresponding transmission) was more dependent on the incidence angle ($\theta$) in the case of the TE mode than for the case of the TM mode. In this case, increasing the angle of incidence up to $\theta$ = 65° allowed us to shift the resonance peak by ~ 1.3 µm, as evident from Fig. 7c. This could be attributed to the fact that the surface plasmons excited in the graphene sheets support the TM mode. It is also worth noting that for the TM mode, in contrast with the TE mode, a larger angle of incidence ($\theta$) results in a wider resonance peak. Based on such an approach, we developed the concept of a graphene-based hyperbolic microcavity for application in the mid-IR.

## Summary

In this work, a novel technique to design a tunable hyperbolic microcavity utilizing GHMMs was proposed and theoretically demonstrated. Further, we demonstrated a scheme for modulating the dielectric thickness in the HMM stack via the use of a triangle wave function. Therefore, the proposed plasmonic architecture allowed us to fully use the structural properties of graphene-based multilayered HMMs as a fundamental element of the active hyperbolic microcavities. The results clearly show that the resonance of the metadevice could be continuously and reversibly switched both with respect to its intensity (up to 30%) and wavelength (up to 2.1 µm) in a controllable manner. Furthermore, our calculations indicated that the graphene-dielectric multilayer structure displays an optical topological transition from an elliptical to a type II hyperbolic dispersion in the mid-IR range, confirming the theoretical predictions from previous works^23,40,51,52^. The practical implementation of our scheme allowed a blue/red shift of the transition wavelength by adjusting the base dielectric thickness or controlling the Fermi level of graphene. The latter is especially effective if accomplished via electrical tuning^53,54^. Hence, shifting the transition wavelength further into the IR can be carried out by adopting the lower chemical potential of graphene or a thicker dielectric. Taking into account the angular reflection spectra, this class of microcavities was sensitive to the incident light angle/polarization, which is crucial in applications such as angular-selective and polarization-dependent plasmonic architectures. It is worth noting that in multi-layer graphene structures is interlayer charge screening effect^55^. However, it can be seen that the carrier density share of the 3rd layer is already quite low, and that of any layer above the 3rd layer is negligibly small^55^. We believe that the concept outlined here provides a novel and promising platform for a variety of applications that depend on a mid-IR energy enhancement and precise spectral switching of the electromagnetic resonances.

## Data Availability

The datasets generated and analyzed during the current study are available from the corresponding authors on reasonable request.
